# Talking about PrEP: South African adolescent girls and young women's communication about pre-exposure prophylaxis with partners, parents and peers

**DOI:** 10.3389/frph.2025.1668275

**Published:** 2025-11-27

**Authors:** Babalwa Sindi, Zoe Duby, Kim Jonas, Kate Bergh, Mari Lotvonen, Catherine Mathews

**Affiliations:** 1 Health Systems Research Unit, South African Medical Research Council, Cape Town, South Africa; 2 School of Public Health, University of Cape Town, Cape Town, South Africa; 3 Department of Psychology, University of Cape Town, Cape Town, South Africa; 4 AYP Programme, Networking HIV/AIDS Community of Southern Africa (NACOSA), Cape Town, South Africa

**Keywords:** adolescent girls and young women (AGYW), pre-exposure prophylaxis (PrEP), HIV prevention, communication, partners, parents, peers, South Africa

## Abstract

**Background:**

High rates of HIV among adolescent girls and young women (AGYW) persist as a critical public health issue in South Africa. Pre-exposure prophylaxis (PrEP) is a promising HIV prevention method for reducing new infections. To enable AGYW to access, make decisions about, and effectively use PrEP, they require support from partners, parents, and peers, which is dependent on effective PrEP communication. This study examined barriers to, facilitators of, and outcomes of effective PrEP communication between AGYW and their partners, parents/caregivers and peers.

**Methods:**

This paper presents data from a qualitative study conducted in seven South African provinces. A total of 68 in-depth interviews were conducted with AGYW aged 15–24 years. Audio recordings of interviews were transcribed and translated into English, and preliminary thematic analysis of transcripts was conducted by a team of analysts. A second phase of analysis focusing on PrEP communication was conducted to further extract meaning from the coded data.

**Findings:**

The barriers to effective communication between AGYW and their partners included a lack of accurate information about PrEP, fear that partners would think that they did not trust them, and stigma related to PrEP's association with promiscuity and PrEP being mistaken for HIV treatment. The barriers hindering PrEP communication between AGYW and their parents were similar, including fear of judgement, and PrEP stigma related to assumptions of promiscuity. Open communication was a facilitator of effective PrEP communication between AGYW and partners, parents and peers. The benefits and outcomes of effective PrEP communication included increased knowledge about PrEP, motivation to use PrEP, PrEP use and continuation on PrEP.

**Conclusions:**

To address barriers to effective PrEP communication, community PrEP awareness campaigns and education programmes are needed and should include men, parents and peers. Increased knowledge and awareness of PrEP is likely to result in reduced PrEP stigma, improved PrEP communication and subsequent uptake.

## 1 Introduction

The Human Immunodeficiency Virus (HIV) continues to be a major global health challenge. According to the World Health Organisation (WHO), about 39.9 million people at the end of 2023 were living with HIV worldwide, with the highest estimated prevalence of HIV (26.0 million) reported in the African region (1). In South Africa, recent studies have shown that the country has the highest prevalence of people living with HIV in the world (2). However, there has been a decline in HIV prevalence from 14.0% in 2017 to 12.7% in 2020, resulting in 7.8 million people living with HIV in the country (3).

Evidence shows that females continue to be more vulnerable to HIV, with prevalence rates of 16.4% compared to 8.8% for their male counterparts (3). Adolescent girls and young women (AGYW) aged 15–24 years have an HIV prevalence almost double that of males of the same age—6.9% for AGYW compared to 3.5% for their male counterparts (4). Various factors contribute to the disproportionate burden of HIV among AGYW including their vulnerability and engagement in sexual risk behaviours, stemming from a lack of communication and support from families about sexual and reproductive health (SRH) and other interpersonal, social, gender related, and structural factors (5). Hence in South Africa, AGYW have been prioritised for oral pre-exposure prophylaxis (PrEP)—a medication that can be taken by someone who is HIV negative to prevent HIV infection—introduced to South Africa in 2016 in a phased manner, initially prioritising population groups at higher risk of HIV acquisition (6, 7).

The initial population group that was prioritised for PrEP was sex workers, which then later expanded to other population groups identified at higher risk of getting infected with HIV, such as men who have sex with other men (MSM) (8, 9). Population groups prioritised for PrEP then expanded to include AGYW, given their HIV risk (7). The PrEP guidelines were updated to include additional individuals at risk of HIV infection such as people with multiple partners, people who inject drugs and individuals who identify themselves as being at higher risk of HIV infection (8). Despite good uptake, with 880,000 people being initiated onto oral PrEP between 2016 and 2023 through the National PrEP programme in South Africa, PrEP continuation remains low, with only an estimated 20% of those who started using PrEP continuing to use it (10). A PrEP trial study conducted in Durban, South Africa found that AGYW women stopped PrEP within the first 3 months of PrEP uptake (11). Amongst the various barriers to PrEP persistence, “PrEP stigma” remains a key challenge and needs to be addressed (12).

In addition to AGYW being considered as a group at higher risk of HIV infection and therefore prioritised for PrEP, this group also requires support and guidance in decision-making around health and behaviour. Adolescence is a period of cognitive development characterised by exploration, experimentation and increased risk taking (13, 14). Sexual risk behaviour at the adolescent stage has been identified as a major public health challenge (15). Further, adolescents are strongly influenced by peer groups and peer pressure (16). Research shows that adolescent girls' behaviour and decision-making are easily influenced by their peers, and that adolescents tend to get involved in risk behaviours with people same age as them than alone (17).

Communication is defined as the process of passing a message to make meaning between two or more individuals (18). Poor communication can result in problems which could be avoided through effective communication (18). Poor communication about sexual and reproductive health (SRH) between AGYW and their parents is associated with negative SRH outcomes, including increased risk of HIV (19). However, evidence suggests that there are barriers in communication between AGYW and their mothers or caregivers (19). For AGYW to make informed decisions that lead to positive SRH outcomes such as protecting themselves from HIV, delaying pregnancy and their health in general, they need to talk to their partners and parents about SRH (19).

Communication about health for youth is an important factor in ensuring their sexual and reproductive health and wellbeing (14). For AGYW to be able to make decisions about PrEP use and successfully access, use and adhere to PrEP, they require support from partners, parents and peers, which is dependent on effective PrEP communication. By using the term “PrEP communication”, we refer to AGYW's discussion about PrEP. This includes discussions with people that AGYW talk to about PrEP, PrEP use experiences and decisions/intentions to use PrEP. Evidence from Sub-Saharan African research shows that it is crucial for parents and adolescents to communicate effectively as this could play a role in addressing misconceptions and enable a supportive environment for adolescents to feel comfortable in discussing sexual health related topics (20).

Through the analysis of data collected from AGYW residing in communities in which a large combination HIV prevention programme, providing oral daily PrEP, was implemented, this study aimed to identify and examine the barriers, facilitators, and factors that influence PrEP communication and the effects of PrEP communication among AGYW in South Africa.

## 2 Methods

Data presented in this paper were collected between March and May 2024 as part of an evaluation of a combination HIV prevention programme for AGYW in South Africa. Data collection took place in seven South African provinces: Eastern Cape, Western Cape, Free State, Mpumalanga, Northwest, KwaZulu Natal and Gauteng. This study used qualitative methods to explore factors that influence PrEP communication among AGYW, and the effects of PrEP communication among AGYW.

### 2.1 Study population

The study population included AGYW between 15 and 24 years from communities in which the combination HIV prevention programme had been implemented. Participants were recruited at their homes as part of the parent study household survey. Those AGYW who participated in the survey were asked if they wanted to participate in a follow up interview. We then conducted telephone interviews with the survey participants who indicated they were willing to be interviewed. As seen in Table 1, a total of 68 AGYW were interviewed in the qualitative study, 13 AGYW aged 15–17 years, and 55 AGYW aged 18–24 years).

**Table 1:** Study sample.

Province	15–17 years	18–24 years	Total
Eastern Cape	1	2	3
Free State	5	6	11
Gauteng	2	6	8
KwaZulu Natal	0	7	7
Limpopo	0	0	0
Mpumalanga	0	14	14
Northwest	2	8	10
Western Cape	3	12	15
Total	13	55	68

### 2.2 Data collection

The study used an interpretivist paradigm. Qualitative in-depth telephone interviews were conducted by trained researchers with experience in qualitative interviewing. Interviewers were all female with tertiary education, and spoke the study languages: isiXhosa, English, Setswana, isiZulu, Sesotho and Afrikaans. Participants were interviewed in the language of their choice at their preferred time. To ensure confidentiality and privacy, participants were asked to be alone in the room they were in before the interviews. The interviewers also checked to make sure that participants felt comfortable in their environment before starting the interview. In addition, interviewers checked participants comfort throughout the interviews. Telephone interviews were recorded using MS Teams software and the participants gave consent for the audio recordings. Each interview took approximately 1 h in length and followed a semi-structured interview guide. The recorded audio files were saved in secure password protected servers and labelled with unique, anonymised identification numbers which were allocated to each participant. Participants were informed of this during the consenting process.

### 2.3 Data processing and analysis

Data analysis was iterative and conducted in three stages. In the first phase, the whole data set was analysed where audio recordings of the interviews were directly translated into English from original languages in which the interviews were conducted and saved with participant's PIDs. Quality control and verification of data integrity procedures were performed by interviewers and data managers. Thematic analysis was conducted using raw data from interview transcripts and followed a process of grouping data and organising it into meaningful patterns. The study used both deductive and inductive analysis by searching, organising, mapping, creating codes and themes to make meaning of the data using a summary memo. For deductive analysis, authors developed codes and themes using the interview guides. After determining prior themes, analysts thoroughly went through all the transcripts to find matching data that support the pre-determined themes. In addition, memoing was used in the process of developing codes and themes and extracting meaning from the data. Coders and analysts for the original study were ZD and BS. ZD reviewed the coded data to ensure coder agreement. A second phase of analysis focusing on PrEP was conducted to further extract meaning from the coded data. Subsequently, a deeper analysis was conducted focusing on PrEP communication. For this paper, inductive analysis was conducted by BS under ZD guidance, while KJ, KB, ML and CM reviewed the themes. BS and ZD familiarized themselves with the data through repetition reading from analytic summary memos. After repetitive reading as the process of familiarization with data, we then created codes and themes by grouping together similar data, repeatedly revisiting the data and refining the themes.

### 2.4 Ethical considerations

Ethical approval for this study was received from the South African Medical Research Council Ethics Committee (HREC REF: EC027-8/2023). The study was also approved by the Department of Health from each province. Participants were provided with information about the study and voluntarily took part. Informed consent processes were conducted in the language preferred by participants. Verbal consents were obtained by the trained interviewers, and audio recorded. During the interviews, confidentiality and anonymity were maintained by using a unique study participants numbers for each participant. Participants were informed that study participation was voluntary, that there were no wrong or right answers and that they may withdraw anytime. Participants above the age of 18 years consented for themselves in a form of verbal consent and for those under 18 years, verbal consents from parents, caregivers or guardians were obtained first then they provided assent for themselves. To obtain verbal consent for AGYW, we asked to audio-record them and asked them to state their names and that they agree to participate in the study. And for parents we followed a similar procedure: they were asked to state their names and that they agreed to their daughter's participation in the study, followed by the daughter's names. These short audio-recordings were stored separatly from the interview recordings. Participants received R200 (approximately US $11) reimbursement in the form of a grocery voucher for taking part in the study and households were reimbursed with R50 (US $3) in a form of electricity or airtime or cell phone data.

## 3 Findings

This section focuses on themes that emerged related to discussions and communication that participants had about PrEP with their partners, parents and peers, including barriers, facilitators, and outcomes of PrEP discussions and communication. Quotations from English transcripts are shown in italics text, followed by participants' details (age group and Province) in brackets.

### 3.1 Barriers to PrEP communication

#### 3.1.1 Prep stigma

A common theme related to barriers to PrEP communication with partners was the fear that PrEP is a sign of lack of trust. Participants described the challenges they faced in discussing PrEP with their partners, often as a result of fear of disproval and fear driven by an anticipation of being judged. Participants worried that their partners would disapprove of PrEP if they discussed it with them. Another reason cited for not discussing PrEP with partners was the fear that male partners will perceive them as being disloyal. Anticipating partners' reactions could make AGYW reluctant to talk about PrEP with their partners.

The participant's response below illustrates some of the relationship level barriers to AGYW discussing PrEP with their partners, such as perceptions of trust.

(What could make it difficult for AGYW to discuss PrEP with their partners) It could be that these partners could think that this one is unfaithful. I too would be sceptical…Why would you want to protect yourself if we are people that test and know our statuses? (AGYW 18–24, North West)

Another participant who shared that she never discussed PrEP with her partner, indicated her willingness to take PrEP but was reluctant to communicate that with her partner due to her fear that he might regard her PrEP use as an accusation that he has HIV.

No (I have never talked about PrEP with my partner)…He will tell me he is not sick (HIV positive)…He will think I am accusing him of being sick. (AGYW 18–24, Mpumalanga)

The narrative from the participant below describes how she discussed PrEP with her partner, but was met with his disapproving reaction.

Yes (I have discussed PrEP with my partner)…Aye! he was not fine, he is horse (Whore, slang for sexually promiscuous) he also knows…he is a whore…mm it was difficult (to discuss PrEP with my partner) because he did not approve…he did not approve at all, I don't know why he did not approve, I don't know whether he wanted to infect me or what he wanted to do. (AGYW 18–24, North West)

Also related to PrEP Stigma, was the sub-theme that PrEP is associated with promiscuity. One participant commented that she has never talked about PrEP with her partner even though she was using it, due to the fear that he might think that she sleeps around.

Ah-ah (No I have not discussed PrEP with my partner while I was using PrEP)…I was scared that he would ask me questions whether I'm sleeping with other people or not, you see…He doesn't know anything (about PrEP). (AGYW 18–24, North West)

Another type of PrEP stigma related to people confusing PrEP with antiretroviral HIV treatment due to lack of knowledge and information about PrEP. AGYW explained that they do not discuss PrEP with partners because of this anticipated stigma and fear that they will be assumed to be HIV positive if they take PrEP, due to the common mistaking of PrEP for antiretroviral treatment, as illustrated in this participant's narrative:.

No (I have not talked about PrEP with my boyfriend)…Because he will think that I have HIV now that I am taking pills every day. (AGYW 18–24, Mpumalanga)

The quotation below shows that AGYW's own feelings of lacking sufficient information about PrEP also serve as a barrier to discussing PrEP with partners.

No, I haven't (discussed PrEP with my partner)…that is because I don't have much information on PrEP to talk about it…I have tried to talk to someone about PrEP…(I would consider using PrEP) if it were to be explained to me properly by someone who is professional. (AGYW 18–24, Mpumalanga).

The narratives from the participants show that PrEP stigma also negatively impacts AGYW's ability to discuss PrEP with parents. A key theme related to barriers to PrEP communication with parents described by AGYW was fear of judgement by parents. Some participants indicated that they do not discuss PrEP with their parents due to fear of the stigma and judgement that might arise, as evident in this participant's comments:

Ah-ah No (I have not discussed PrEP use with my parents)…I was scared that they would judge me or something. (AGYW 18–24, North West)

Concerns that parents would associate PrEP with promiscuity served as a barrier to communication. Some AGYW reported not talking to parents about PrEP because of the previous reactions that they have received from their parents. Participants articulated the belief that talking about PrEP with parents might lead them to believe that their daughter is motivated to discuss PrEP with them because she sleeps around.

It's difficult (to talk about PrEP to my parents)…Judging from how I know them they might have this perspective that I go sleeping around and that's why am taking PrEP. (AGYW 18–24, Gauteng)

A self-perceived lack of information about PrEP also prevented PrEP communication. A participant mentioned that a barrier she faces in talking with parents about PrEP is not having enough information about PrEP. She feels that she would need to be better informed about PrEP before she speaks to parents.

No (I have not discussed PrEP with my parents)…if I could do more research about PrEP so that when I go to them to speak about PrEP I am better prepared with more information. (AGYW 18–24, Gauteng)

The narrative below demonstrates how challenging it is for young people to discuss sex related topics with parents. Talking about PrEP would indicate that they are sexually active and AGYW might be embarrassed to discuss such matters with parents. Some AGYW believed that it is better when it is the parent who introduces the topic of PrEP.

No (I have never discussed PrEP with my parents/caregivers)…I become shy…I think if they introduce the topic then I can discuss it with them…if you try to discuss it (PrEP) with them they will start asking you questions like why do you want to know about these pills?…I think if they can tell me about PrEP…then I will be able to discuss it with them. (AGYW 18–24, Free State)

However, some AGYW said they were able to communicate about PrEP with parents, but their narratives suggested that in doing so they were themselves sharing inaccurate information about PrEP. One participant indicated that she did talk about PrEP with her parent, but told them it was also a contraceptive, demonstrating that without being sufficiently informed about PrEP, communication about it can also spread inaccurate information.

Yes (I have discussed PrEP with my parents)…They (my parents) were surprised…I'm able to discuss anything with them, so I was telling them about PrEP and that it also prevents pregnancy…it was easy (to discuss PrEP with my parents)…it is because they like us to sit down and discuss health issues. (AGYW 18–24, Free State)

Participant narratives expressed that AGYW also face challenges in discussing PrEP with their peers driven by judgement and peer power dynamics.

Yes (there are people I chose not to discuss PrEP)…They are my peers who like to say we are self-centred and we like doing as if we know about health…so I decided to leave them (peers)…because if you discuss with them some will listen and others won't. Those who don't listen will say something out of order, and that is not right. (AGYW 18–24, Free State)

### 3.2 Facilitators to PrEP communication

#### 3.2.1 Open communication

Regarding facilitators for communicating about PrEP with partners, open communication was described as important. One participant highlighted the importance of maintaining open and free communication with a partner in a sexual/romantic relationship makes it easy to talk about PrEP.

(What made it easy to discuss PrEP with my partner)…Me and my partner am talking about my last year relationship, we are people that are free about everything, we do not hide anything and even these programmes I did tell him that am on a PrEP study, I do not know how to tell you but we open to each other about everything. (AGYW 18–24, North West)

Similarly with partners, a key facilitator to PrEP communication was parents' openness to communication. Narratives from participants who talk about PrEP with their parents indicate that parents' openness to communication and expressed interest combined with PrEP knowledge facilitated conversations about PrEP:

Yes (I have spoken with my mother about PrEP)…I asked questions like “why do most people use it?”…She told me they use it because they don't want to get infected….Yes (it was easy to talk to her about PrEP)…because she always asks me what do I learn from school and all those stuff. (AGYW 15–17, North West)

Openness to discussing SRH topics also facilitated conversations about PrEP, as described by a participant who reported that she finds it easy to talk about PrEP with her mother because her mother talks about HIV with her.

Yes my mom knows about it (PrEP)…that is a good thing because there is something that protects us from being sick…My mom is one person that likes to talk about HIV saying you…“if it happens that one of you have HIV it's better for one to take the medication and accept oneself”, so it's something that makes it easy for me to tell my mom because it's something she talks about at home. (AGYW 18–24, North West)

Likewise, another AGYW mentioned that being able to discuss contraceptives with parents also makes it easy to talk about how one can protect herself from STIs and HIV.

(What makes it easy to discuss PrEP with my parents)…If you can freely speak to you parent about contraceptives, obviously it should also be easy to talk to them about sexual transmitted diseases on how you could protect yourself and what you can use. She would be the one that tells you that, “my friend is using such, why don't you also use such a thing?” (AGYW 18–24, North West)

#### 3.2.2 Relatability

It was suggested that a sense of relatability and mutual understanding between peers makes it easy to discuss PrEP. One participant reported that she talks about PrEP with her peers and they even joke around it which suggests that shared humour also makes AGYW are more comfortable to talk about PrEP with peers.

Yes, we (my friends and I) do talk about it (PrEP) until it becomes a joke…The pills are big so, if you drink them you will choke, they prefer an injection rather than pills…So, it ends up being a joke, some say you need to crush them before drinking them…things like that…they do (understand PrEP) but not that much but they do know what PrEP does. (AGYW 18–24, Mpumalanga)

Relatability also linked to being of the same age as peers, which can play an important role in facilitating conversations about PrEP with peers because they have similar interests and beliefs. Being the same age allows AGYW to discuss health related topics freely.

No (I have never talked about PrEP to my partner/boyfriend)…Yes (I have talked to my friends about PrEP)…Most of the time it's them that initiate such conversations (about) PrEP, because they visit the clinics more regularly so they see people that deal with such things…when they go to the clinic they normally meet those people so they engage about such things way more than me…(It's easy to discuss PrEP with my friends) because we're in the same age group and we are used to speak about such things to each other…who love to know about new things…especially about things that concern their health. (AGYW 18–24, Gauteng)

### 3.3 Outcomes of PrEP communication

The participants' narratives suggested that PrEP communication among peers increases PrEP knowledge. AGYW shared their own experiences of talking about PrEP with their peers, and described the way in which communication about PrEP helps to share information with people who have no knowledge about it, and those who have not heard about PrEP before. Those peers who had knowledge helped to share the information with those who did not know PrEP. However, not all peers showed an interest in talking about PrEP.

Yes (I have discussed PrEP with my friends/peers)…Others were surprised as they heard about it for the first time and others already knew about it…Those who knew about it helped to share the information with those who did not know, and they were surprised and went to get PrEP…(AGYW 18–24, Free State).

A similar view was demonstrated by another AGYW who first heard about PrEP from friends and suggested that AGYW motivate each other. The narrative also indicates that some AGYW from this group of friends knew nothing about PrEP and first heard about it from the discussions they had.

My friends, I did discuss with them about those things (PrEP)…All of them…the same way as I was surprised when I was told about it, they were also surprised because they didn't know there's something that prevent HIV, other than testing…(What could make it easy/difficult to discuss PrEP with my friends)…It is something easy because we discuss about…I mean we discuss about things a lot…such as when you have symptoms of HIV, what you should do or when you symptoms for TB or when you prevent yourself during sex…Yes, those are the things we can talk about most of the time…I feel good (discussing PrEP with my friends) because I'm able to encourage them…When something is bad, I tell them that this thing is bad. (But) for me you see with those pill (PrEP)…I felt good when I told them (my friends) about them (PrEP), so that they can realize what they should do, they need to check if they can take them, or they should go get tested (for HIV), they should choose between those two things. (AGYW 18–24, Free State)

A sub-theme under the benefits of PrEP communication was related to the suggestion that PrEP communication among peers increases PrEP use/intention to use PrEP, as illustrated below.

(I have talked about PrEP)….I talked to my friend…She felt good and she wanted to go and get it. (AGYW 18–24, KwaZulu Natal).

Another participant demonstrated that discussing PrEP with friends serves as a motivation for other peers to use PrEP. This is demonstrated below in the narrative of a participant who even shared PrEP with her friends out of her desire to keep them safe and healthy:

(Yes I have discussed PrEP with my friends/peers)…They also wanted it, I even gave them mine, they would always come to me to take them, just because they did not want to get sick…I did not want to bury anyone, I was just telling them what should we do. (AGYW 18–24, North West)

Another participant agreed that talking to her friend about PrEP motivated the friend to start using PrEP, suggesting that PrEP discussion between peers serves a motivation for others use PrEP.

I talked to my friend (about PrEP)…She felt good and she wanted to go and get it. (AGYW 18–24, KwaZulu Natal)

Similarly, a participant pointed out that talking to her friends about her own PrEP use inspires and motivates peers to use it:

I would disclose (I was using PrEP) to the next person and explain it to him/her…It could be my friend so that she can protect herself because there is no one that could tell you that they have this disease or are infected with it…they should also help themselves as I also have helped myself too…Yes we (friends/peers) have spoken about PrEP…I feel happy because they also wish to access it. (AGYW 15–17, Free State)

Narratives from participants who talk about PrEP with their parents indicate that talking about PrEP with parents makes it much easier for them to use PrEP, especially when parents/caregivers are encouraging of PrEP use.

When I came home with the (PrEP) pills my mom was happy saying that at least there are pills that prevent this virus (HIV) unlike having it in your body forever, that would not be nice. (AGYW 18–24, North West)

The ability to speak openly about PrEP with parents' and caregivers', especially those whose reactions were positive and supportive of PrEP use, creates an enabling environment for AGYW to adhere to and continue using PrEP:

Yes (I have talked to someone about PrEP), I have. My aunt…Her reaction was fine, she said PrEP was alright…She said if I take them, I should be able to drink them every day and not stop because PrEP is a prevention pill. (AGYW 18–24, Mpumalanga).

## 4 Discussion

To the authors’ knowledge, this is the first study to focus specifically on communication about PrEP between South African AGYW and their partners, parents and peers. Participants' narratives about PrEP communication with their partners, parents and peers provided valuable insights into barriers, facilitators and benefits of effective communication about PrEP. The findings from this study show that there are several barriers to South African AGYW's effective PrEP communication with partners and parents about PrEP. Barriers described included PrEP stigma related to PrEP's association with being promiscuous and being HIV positive (for partners in particular), a fear that talking about PrEP demonstrates a lack of trust in partners and a fear of judgement from parents. The positive outcomes of PrEP communication included increased PrEP knowledge and intention to use and continue using PrEP. However possible negative outcomes included judgement from parents, and disapproval or accusations of being unfaithful from partners. AGYW described that communicating with peers about PrEP was much easier than with parents and partners. PrEP communication facilitators with both partners and parents include openness to communication and PrEP knowledge for parents. Relatability was also described as a facilitator to PrEP communication between peers. The benefits of being able to communicate effectively about PrEP were described as increased PrEP knowledge, increased PrEP use, increased intention to use PrEP and motivation to use/continue using PrEP for AGYW.

One of the key findings indicates a barrier for PrEP communication between AGYW and their partners relates to their fear of being accused of lacking trust in their partners. The findings demonstrated that AGYW face challenges in raising discussions about the topic of PrEP with partners. The main cause of this is the fear that the male partner will accuse his girlfriend of suggesting that he has HIV. Findings from a study conducted in Kenya, Cape Town and Johannesburg among AGYW show that partners are the only group with a strong negative influence including lack of support for or disapproval of AGYW's HIV prevention choices (21). Our study also found that, according AGYW's narratives, partners have strong negative influence on AGYW's HIV prevention options. Our analysis demonstrated that fear of judgement experienced and anticipated by AGYW from partners was a challenge to PrEP communication between AGYW and their partners. Findings from previous studies demonstrate that one barrier to disclosing PrEP use to partners centred around concerns of accusations of disloyal or unfaithful (12). The fear makes AGYW refrain from initiating PrEP discussions with their partners. Sub-Saharan African studies show that partners play a crucial role in adult women's daily oral PrEP pill taking (22), suggesting that partners' effective communication could play an important role in supporting AGYW PrEP uptake and adherence.

Participants also narrated that it is difficult to talk to partners about PrEP due to issues related to partners' disapproval of PrEP. These narratives illustrate underlying issues of relationship power dynamics which affect AGYW's agency and decision-making in those relationships. Evidence from previous research has argued that in spite of shifting gender norms, unequal power dynamics still persist in heterosexual/“straight” relationships amongst adolescents and young people in South Africa (23). In the South African context even, condom use in a relationship is influenced by social gender norms (24). These power dynamics in relationships are influenced by societal norms, as identified in prior research suggesting that social cultural norms influence sexual behaviour and decision making within Sub-Saharan Africa, and that adolescents are more likely to talk about SHR with their mothers than fathers (25). The inability to discuss PrEP with partners is a challenge for AGYW because lack of communication about PrEP exposes AGYW to higher chances of HIV infection. Effective communication between partners could reduce the risk of HIV infection because being able to talk about PrEP means taking an informed decision together.

Some participants demonstrated that they find it difficult to raise the topic of PrEP with their parents because of the fear that they will be judged. Participants shared that they would prefer their parents to initiate conversations about sex related topics, whereas those participants who had mothers who discussed SRH topics with them found it easy to discuss PrEP with them. An intervention study conducted in Cape Town demonstrated that participants experienced challenges in explaining PrEP to their mothers influenced by misinformation that PrEP is for sick people or HIV treatment or would lead to the girls acquiring HIV (12). An additional barrier for PrEP communication between AGYW and their parents was the fear that PrEP would be associated with promiscuity and being sexually active. In support of this, a study on experiences of PrEP disclosure found that after participants disclosed to their fathers, some reported PrEP stigma related to beliefs about PrEP promoting promiscuity (12). Evidence from previous research indicates that participants reported a link between PrEP disclosure and stigma associated with HIV and sexual activity (26). In some instances, disclosing PrEP use to partners and parents could result in negative outcomes such as discrimination and violence, and hence it is important for AGYW to be supported and guided if they choose to disclose (22). A key challenge to PrEP communication with parents can be linked to evidence showing that South African mothers' reluctance to talk about sexuality is associated with a lack of effective communication, knowledge and cultural norms (19). Fear that discussion will motivate sexual behaviour is also a contributing factor in mothers' resistance to talk about PrEP (19). Additionally, AGYW's fear to talk about PrEP with parents might be caused by not being familiar with discussing sensitive topics with their parents. Research evidence shows that parents feel uncomfortable to talk about sensitive topics with their children and often shift the responsibility to other family members (27). These barriers to sexuality communication between AGYW and their parents need to be overcome, because AGYW need to talk to parents for support and advice in order for them to make informed decisions with regards to their sexual and reproductive health.

Another barrier to PrEP communication with parents was a lack of information about PrEP. Participants in this study indicated that not having enough information about PrEP themselves made it difficult to have conversations about PrEP with parents. Participants in our study expressed a need to be prepared with the necessary information about PrEP themselves before raising the topic of PrEP with parents. This is compounded by the fact that AGYW indicated that some parents also do not have information on PrEP and therefore have misconceptions about it. Evidence shows that it is common for parents to mistake PrEP for other medications, such as HIV treatment or pills for abortion (12, 28). The lack of information hinders PrEP communication between AGYW and their parents. In addition, some AGYW indicated that they were able to communicate about PrEP with parents, but their narratives indicated they were sharing inaccurate information. This illustrates the critical need for programmes and interventions to provide accurate information on PrEP to both parents and AGYW. In addition, our findings show that judgement and peer power dynamics between AGYW and their peers makes it difficult for AGYW to talk about PrEP among themselves.

AGYW explained that parents that show an interest in talking to their daughters enable AGYW to raise sensitive issues such as PrEP in discussions with their parents. In support of this, previous evidence shows that open communication is crucial in building good rapport with teenagers (29). In addition, discussions between parents and children are seen as a positive factor influencing SRH behaviour choices, although these discussions are often uncomfortable, driven by fear and unidirectional (14). Improving parents' levels of awareness and information about PrEP can lead to more effective communication about PrEP between AGYW and their parents and play a huge role in making sure that AGYW make healthy decisions through support and parental involvement. In this way, AGYW will talk freely with parents about PrEP and increase PrEP uptake. Also, misinformation about PrEP can be dispelled if parents are also involved and supportive. Making efforts to include parents in PrEP promotion and education could improve PrEP uptake and acceptability (28). Evidence has shown that successful ways for involving parents and getting their acceptance and support for AGYW PrEP use have included building relationships through school-based parents' meetings (28).

A healthy sexual or romantic relationship entails being able to openly communicate with each other. Findings from this study indicate that AGYW in relationships where the partners are open to communication find it easier to talk to their partners about PrEP. Participants demonstrated that talking about everything with their partners made it easy for them to talk to their partners about PrEP. In support of this, research on PrEP disclosure shows that benefits of disclosure to partners is linked to motivating discussions around HIV prevention such as HIV testing between partners and providing support such as reminders to take pills (12). Communication between partners has been studied as an element that creates emotional bonds between partners which allow them to talk about everything including sex related topics including PrEP (30).

Most of the participants in this study spoke about PrEP with their peers. Young people are more comfortable to talk about sex related topics, including PrEP, with their peers/friends compared to talking with parents. Evidence shows that adolescents are more influenced by peers than parents and family (31). In addition, adolescents are more likely to adopt their peers' behaviour (32). A previous PrEP study reported that having friends also taking PrEP also made it easier for participants to talk about things and remember to take PrEP pills (12). This suggests that AGYW can influence each other positively and mutual experiences make it easy for AGYW to talk about PrEP amongst each other which could be of benefit to the roll out of other PrEP modalities even though some issues relating to communication may differ.

This study demonstrates the benefits of being able to communicate effectively about PrEP. PrEP communication is a crucial component in spreading knowledge about PrEP, increasing PrEP use/intentions to use PrEP and supporting PrEP use continuation. Evidence from a study conducted in South Africa and Zimbabwe in 2020 shows that participants viewed disclosure as an important factor in PrEP adherence because it allowed them to take their PrEP freely even in the presence of the friends and friends provided support instead of reacting negatively (26). Existing literature shows that availability and accessibility of ARVs has lowered the concern of HIV infection (31). In contrast AGYW's narratives from this paper indicated that telling friends that one is taking PrEP motivates the peers to also start using PrEP themselves to protect themselves, driven by the fear of sickness and death. This point is also discussed in the literature in that adolescents are easily influenced by peer groups in terms of behaviours, attitude and decisions (16). This highlights that promoting peer to peer education on PrEP could be an effective way of enhancing PrEP uptake in South Africa.

AGYW in this study demonstrated that PrEP discussions with their parents motivate and support them to use, and continue using PrEP. A previous study conducted in South Africa and Zimbabwe indicated that positive PrEP disclosure to family, friends and sexual partners were associated to improved PrEP uptake for participants who effectively explained PrEP and its benefits as those involved ended up providing support in a form of reminding the participants to take their daily pills (25). Another study conducted in the United States argued that mothers play a role in reminding AGYW to use their contraceptives and mailing prescriptions for contraceptives to their children (33). Similar results were discovered in an intervention study conducted in Cape Town: some participants reported receiving support from their mothers in a form reminder to take pills (12). These results were echoed in our study where AGYW explained that parents created an enabling environment for AGYW to use or continue using PrEP. This indicates that effective communication can enable parents to remind AGYW about their PrEP appointments dates and taking pills. A recent study also found that lack of support from mothers to AGYW is seen as negatively affecting PrEP adherence (21). Hence, effective communication about sexual reproductive health related topics and PrEP between parents and their adolescents could support PrEP use and adherence.

## 5 Limitations

Due to the scope of the study, the data is limited to the narratives of AGYW and does not include the perspectives of other stakeholders. Parents, peers and partners were not part of the study and were not able to provide their insight on PrEP communication, which would be a topic worthy of further research. In addition, religious beliefs were not explored as part of barriers or facilitators of PrEP communication in the study, which could be explored in future studies. Data relating to PrEP communication emerged mostly from participants between the ages of 18–25 years, and in interviews in the Northwest province so the findings may not represent the views of other provinces equally. Therefore, the sample size does not allow for comparison between the age groups; future research should unpack the differences between adolescents and young women. It is also important to note that the findings in this paper relate to oral daily PrEP, as that was the only PrEP modality offered to AGYW by the programmes in the study communities. Some of the issues relating to communication about PrEP are likely to differ for other PrEP modalities, such as injectables, as issues around stigma and disclosure to parents may differ.

## 6 Conclusions and implications

The findings from this study are valuable for HIV prevention efforts in South Africa as they provide information on the barriers to and facilitators of PrEP communication between AGYW and their parents, partners and peers, demonstrating the importance of open and effective communication in PrEP uptake and adherence. The authors were not aware of any prior studies focusing specifically on AGYW PrEP communication with parents, partners and peers. Findings demonstrate that it is not easy for some participants to talk about PrEP with their parents and partners due to the stigma associated with PrEP. This suggests a need for policies and programmes providing PrEP to AGYW to include educational campaigns to inform and educate men, boys, parents, peers, and communities about PrEP and SRH. Such educational campaigns could help to raise awareness, address misconceptions, and ensure positive framing of PrEP, thus increasing demand creation, reducing PrEP stigma, supporting AGYW to communicate about PrEP and disclose PrEP use, therefore creating an enabling environment for AGYW PrEP use (25). The inclusion of PrEP in the curriculum provided during Life Orientation lessons at schools in South Africa would also help to ensure that the information is accessible to both boys and girls, catering for all genders and relationship types. Improving levels of PrEP knowledge will facilitate PrEP communication and AGYW's ability to discuss PrEP and SRH related topics with their parents and partners, thus enabling more informed sexual decision making and agency to enact HIV prevention behaviors.

## Data Availability

Publicly available datasets were analyzed in this study. This data can be found here: https://www.samrc.ac.za/intramural-research-units/HealthSystems-HERStory.
