# Supplementary material for: Talking about PrEP: South African adolescent girls and young women's communication about pre-exposure prophylaxis with partners, parents and peers
Source: Front Reprod Health. 2025 Nov 27;7:1668275. doi: 10.3389/frph.2025.1668275 (PMC12695815; doi:10.3389/frph.2025.1668275)
Supplement: Supplementary file 1 [file Datasheet1.pdf]

HERStory3 Qual

FOCAL AREA: PrEP

Code Tree

<u>AGYW PrEP Knowledge and Information</u>

- **<u>AGYW Knowledge about PrEP</u>**
    - <u>No knowledge of PrEP</u>
    - <u>Some knowledge of PrEP</u>
- <u>Sources of Information about PrEP</u>
- **<u>AGYW PrEP Decision Making</u>**
    - <u>Decision not to use PrEP</u>
    - <u>AGYW Intentions to Use PrEP</u>
- **<u>AGYW PrEP Communication</u>**
    - <u>Discussing PrEP with parents/family</u>
        - <u>Have discussed PrEP with parents/family</u>
        - <u>Have not discussed PrEP with parents/family</u>
    - <u>Discussing PrEP with partners</u>
    - <u>Have discussed PrEP with partner</u>
        - <u>Have not discussed PrEP with partner</u>
    - <u>Discussing PrEP with friends/peers</u>
        - <u>Have discussed PrEP with friends/peers</u>
        - <u>Have not discussed PrEP with friends/peers</u>
    - <u>AGYW perceptions on Community Views on PrEP</u>

<u>PrEP Journeys</u>

- **<u>PrEP Journeys</u>**
    - <u>PrEP Journeys - currently using PrEP</u>
    - <u>PrEP Journeys - discontinued using PrEP</u>
    - <u>PrEP refusers: decisions not to use PrEP</u>
        - <u>Intentions to use PrEP in the future</u>
