# Supplementary material for: Talking about PrEP: South African adolescent girls and young women's communication about pre-exposure prophylaxis with partners, parents and peers
Source: Front Reprod Health. 2025 Nov 27;7:1668275. doi: 10.3389/frph.2025.1668275 (PMC12695815; doi:10.3389/frph.2025.1668275)
Supplement: Supplementary file 2 [file Datasheet2.pdf]

HERStory 3 PrEP Questions from AGYW Interview Guide

PrEP

Now let's discuss PrEP

- **(for all AGYW)** What do you know about PrEP?
    - Where did you hear about PrEP from?
        - Probe: social media, other media (TV, radio etc), peers, influencers, teachers, safe spaces, mobile clinics, parents/caregivers, community members, other
        - What did you hear about PrEP?
    - Were you ever offered PrEP in the programme?
        - What were you told about PrEP when you were offered it?
        - What were your feelings and thoughts about PrEP at the time?
            - Positive feelings
            - Negative feelings
        - Have these feelings changed over time – why / how?

- **(for HIV negative AGYW)** Are you currently using PrEP?
    - **If yes**, could you tell me about your experience using PrEP?
        - What made you decide to take PrEP
            - Was there anyone who helped you to make this decision?
        - How do you feel about using PrEP?
            - What things do you like about it?
            - Are there things you don't like about it?
            - Do you plan to continue using PrEP?
                - What would make you want to continue using it?
                - What would make you want to stop using it?
        - Could you tell me about any discussions you've had about using PrEP.
            - Have you discussed this with your partner, if you have one?
                - If yes, what was their reaction?
                - If no, are there reasons you haven't discussed it with them?
                - What makes is easy or difficult to discuss your PrEP use with a partner?
                - What would make it easier?
            - Have you discussed this with a parent or caregiver?
                - If yes, what was their reaction?
                - If no, are there reasons you haven't discussed it with them?

            - What makes is easy or difficult to discuss your PrEP use with parent or caregiver?
            - What would make it easier?
        - Have you discussed this with peers/friends?
            - If yes, what was their reaction?
            - If no, are there reasons you haven't discussed it with them?
            - What makes is easy or difficult to discuss your PrEP use with a friend?
            - What would make it easier?
        - Are there people you have chosen not to discuss your PrEP use with?
    - Where did you get PrEP from?
        - How was your experience getting it?
        - What was the person who gave it to you like?
        - How did you feel at the time you were getting it?
        - Are there things that made your experience good?
        - Are there things that would have made your experience better?

- **If you have been offered PrEP but are not using it**, could you share with us the reasons you have decided not to use it?

- **For those not using PrEP**, could you tell us about any experiences you've had discussing PrEP with other people?
    - Probe:
        - Parents / caregivers
        - Friends / Peers
        - Partners
        - Anyone else
